# Use of concentric linear velocity to monitor flywheel exercise load

**DOI:** 10.3389/fphys.2022.961572

**Published:** 2022-08-12

**Authors:** Fernando Martín-Rivera, Marco Beato, Vicente Alepuz-Moner, Sergio Maroto-Izquierdo

**Affiliations:** ^1^ Research Group in Prevention and Health in Exercise and Sport, University of Valencia, Valencia, Spain; ^2^ School of Health and Sports Sciences, University of Suffolk, Ipswich, United Kingdom; ^3^ Institute of Health and Wellbeing, University of Suffolk, Ipswich, United Kingdom; ^4^ Department of I+D+I, Ionclinics and Deionic, Valencia, Spain; ^5^ Department of Health Sciences, European University Miguel de Cervantes, Valladolid, Spain; ^6^ Proporción A, Applied Sports Science Centre, Valladolid, Spain

**Keywords:** isoinertial, flywheel training, load quantification, load-velocity profile, RPE, encoder

## Abstract

**Purpose:** To propose the concentric linear velocity measurement as a valid method to quantify load and individualise the prescription of flywheel training, we investigated the relationship between inertial load and mean concentric linear velocity (MCLV) during the flywheel squat exercise in a wide spectrum of intensities. In addition, we compared MCLV and subjective rating of perceived exertion (RPE) after each load.

**Methods:** Twenty-five physically active men volunteered for this study (26.5 ± 2.9 years, 179.5 ± 4.2 cm, 81.6 ± 8.6 kg). After familiarization, all participants performed two inertial progressive load tests on separated days to determine the flywheel load-velocity profile and its reliability. Each participant performed 5 set of 6 repetitions of the flywheel squat exercise with different inertial loads (0.047, 0.104, 0.161, 0.245, 0.321 kg m^2^) selected in a counterbalanced and randomized order for each testing day. Average MCLV and RPE for each load were compared.

**Results:** The inter-session intraclass correlation coefficient (ICC) showed values above 0.9 in all the included outcomes (MCLV: ICC = 0.91; RPE: ICC = 0.93). A significant correlation (*p* < 0.01, *R* ^2^ = 0.80) between inertial load and MCLV was found. Similarly, significant correlation models (*p* < 0.01) were observed between RPE and load (*R* ^2^ = 0.87) and (*R* ^2^ = 0.71) between RPE and MCLV.

**Conclusion:** The control of MCLV during flywheel exercise can be proposed as a valid method to quantify load and to individualize the prescription of flywheel training. In addition, RPE responses have demonstrated significant correlations with load and velocity. Therefore, RPE has been proposed as a valid and reliable alternative to control flywheel training.

## 1 Introduction

Muscles are not capable of lifting as much load in the concentric phase as they can lower with control in the eccentric phase due to the well-described force-velocity characteristics of muscles (Douglas et al., 2017). Therefore, loads used during resistance training are limited to those that can be raised in the concentric phase (Wagle et al., 2017). Indeed, resistance training intensity is traditionally prescribed relatively to the maximum concentric strength (based on % of one-repetition maximum [1-RM]) (Suchomel et al., 2016). Thus, a submaximal stimulus is applied during the eccentric muscular contraction of usual weightlifting programs (when no spotters, bands or other equipment are used to provide eccentric overload).

In pursuit of the training process optimization, isoinertial flywheel devices have emerged as an alternative resistance training technology that allows an equivalence in the relative intensity of both concentric and eccentric contractions, while involving the use of the stretch-shortening cycle during resistance training (Tesch et al., 2017; Beato and Dello Iacono, 2020). This non-gravity-dependent technology uses the energy stored in the flywheel system after a maximal concentric action (i.e., inertial kinetic energy that results from the unwinding of the flywheel’s strap) to overload the eccentric action when a brief and concentrated braking action occurs (Maroto-Izquierdo et al., 2019). Flywheel training has been shown changes related to sports performance optimization (Maroto-Izquierdo et al., 2017; Beato et al., 2019; Maroto-Izquierdo and McBride, 2022) and rehabilitation (Wonders, 2019; Maroto-Izquierdo and Nosaka, 2022), injury prevention (Tesch et al., 2017; Monajati et al., 2018), and functional capacity improvements (Tesch et al., 2017; Kowalchuk and Butcher, 2019). Indeed, these brief episodes of eccentric overload induced by flywheel devices and performed at high intensity are associated with greater improvements in both concentric and eccentric force, muscle mechanical power and muscle hypertrophy in healthy and well-trained subjects when compared to those induced by traditional training with free weights and weight-stack machines (Maroto-Izquierdo et al., 2017; de Keijzer et al., 2022). Additional benefits of using a flywheel device is its versatility, including the ability to perform a variety of exercises in different movement planes and force vectors (Gonzalo-Skok et al., 2017), as well as its portability (Suchomel et al., 2019).

Despite the broad benefits reported by flywheel training, one of its main limitations is the difficulty to monitor and adjust training volumes and intensities to fit the needs of each individual (Suchomel et al., 2019). The vast majority of the existing published studies have arbitrarily chosen sets and repetitions (e.g., 4 sets of 7 repetitions) with a given inertial load (ranged from 0.05 to 0.145 kg m^2^) (Maroto-Izquierdo et al., 2017). However, this often leads to “wasted repetitions” (i.e., repetitions needed to spin velocity into the system), unnecessary mechanical stress while neuromuscular fatigue increases and the measures of force and power output decrease, and poor training load management (Suchomel et al., 2019). Inasmuch as it is not possible to prescribe flywheel exercise intensity based on the maximal concentric force capacity, as it occurs in traditional resistance training with the 1-RM (since there is not a maximum load that can be lifted during isoinertial flywheel exercise) (Maroto-Izquierdo et al., 2021). In addition, considering the maximum nature of each concentric contraction during flywheel exercise and its high contribution to the desired effects (Maroto-Izquierdo et al., 2021), practitioners should consider to quantify concentric kinematic outputs during flywheel exercise.

Accordingly, it has recently been shown that, similarly to resistance exercise training (Hill, 1938), concentric and eccentric velocity during flywheel exercise decreased while intensity (i.e., inertial load) increased (McErlain-Naylor and Beato, 2020). Therefore, the control of mean concentric velocity has been proposed to be used an avenue of intensity prescription for practitioners (Carroll et al., 2019). Indeed, the load-velocity profile (LV_profile_) outcome has been shown to be a valid and reliable tool for prescribing flywheel exercise intensity and for assessing training adaptations (Spudić et al., 2020). However, none of the previous studies (Carroll et al., 2019; McErlain-Naylor and Beato, 2020; Spudić et al., 2020; Worcester et al., 2020) that analysed the LV_profile_ during flywheel exercise used an integrated linear encoder to measure kinematic outcomes that might be crucial to describe the kinetic characteristics of flywheel exercise, such as impulse (N s^−1^) (Carroll et al., 2019; Núñez et al., 2020). Notwithstanding, accurate monitoring is quite complicated and required the use of advanced technologies that are not commonly available to practitioners. Rotary encoders, which provide information about the angular velocity of the wheel (Bollinger et al., 2018; Weakley et al., 2019) are widely used for such purpose. However, it should be considered their difficulty to differentiate between concentric and eccentric actions (the wheel only changes its rotational direction between repetitions) and the existence of a dissonance between what happens on the axis (i.e., angular velocity in a conical cylinder flywheel device) (Sabido et al., 2020b; Núñez et al., 2020), and what happens at kinematic level while the participant is performing the exercise. Which in turn has limited the use of velocity for such purposes. Hence, further studies are warranted to determine other feasible load quantification strategies (e.g., linear velocity quantification).

The difficulty to quantify linear velocity during flywheel exercise training has led to employ alternative strategies (Banyard et al., 2019). Several studies have reported a high correlation between the load increase (and its corresponding velocity drop in the LV_profile_) and the subsequent rise in the subjective rating of perceived exertion (RPE) response (Hollander et al., 2008; Hollander et al., 2017). Despite the fact that there is no validated RPE scale for flywheel training, the RPE has been used for assessing both intensity and fatigue during flywheel exercise (Raeder et al., 2016; Timón et al., 2018; Sabido et al., 2020a). Although it is well-known that velocity decrease while inertial load increase during flywheel exercise (Carroll et al., 2019; McErlain-Naylor and Beato, 2020; Spudić et al., 2020; Worcester et al., 2020), these rises in the RPE response not only depend on the LV_profile_, but also on the concentration of blood lactate and many other metabolites (e.g., K^+^,CK). Which in turn, may affect afferent feedback in the central nervous system, decreasing performance and contractile efficiency (Broxterman et al., 2018); and thereafter, influencing the RPE response. Thus, the assessment of the RPE and concentric velocity at different inertial loads might be accurate enough to overpass the aforementioned flywheel monitoring limitations, and thus, optimizing the training process (i.e., avoiding additional unnecessary mechanical stress while maintaining similar measures of force and power output and boosting the recovery process after flywheel training).

So far, no study has described the LV_profile_ during flywheel exercise using the concentric linear velocity by means of a flywheel device integrated linear encoder, either no study has correlated flywheel concentric linear velocity at different intensities with RPE. Thus, to propose the concentric linear velocity measurement as a valid method to quantify load and individualise the prescription of flywheel training, this study aimed to analyse the relationship between inertial load and mean concentric linear velocity (MCLV) during the flywheel squat exercise in a wide spectrum of intensities, as well as to compare MCLV at each load with the RPE in physically active men. We hypothesized that MCLV is a valid and reliable indicator to monitor flywheel training and to determine the load-velocity relationship, as well as MCLV correlates significantly with RPE.

## 2 Materials and methods

### 2.1 Subjects

Twenty-five healthy sports science postgraduate male students volunteered for this study (26.5 ± 2.9 years, 179.47 ± 4.24 cm, 81.62 ± 8.59 kg). All of them had at least 1 year of experience with flywheel training, and no history of neurological disorders or lower limb musculoskeletal injuries. None of them were taking drugs, medications or other substances that could alter their performance during testing. Moreover, participants recorded and then maintained their sleeping, eating, and drinking habits in the 48 h prior to each testing sessions. Participants were informed of the purposes and risks involved in the study before giving their informed written consent to participate. The study procedures were in accordance with the principles of the Declaration of Helsinki and were approved by the local Institutional Review Board (H1421157445503).

Sample size was estimated using the data from a previous study (Sabido et al., 2017) in which the effect of different inertial loads on several kinetic and kinematic variables was investigated for the flywheel squat exercise. Based on the effect size of 0.3 for a possible difference in mean concentric velocity between conditions, it was estimated (*t*-test) that at least 20 participants were necessary for each group, with the alpha level of 0.05 and power (1−β) of 0.80 by G*Power (G*Power 3.1.9.2, Heinrich-Heine-Universitat Dusseldorf, Dusseldorf, Germany; http://www.gpower.hhu.de/). Considering possible dropouts and an estimation error, 25 participants were recruited.

### 2.2 Procedures

**FIGURE 1:**
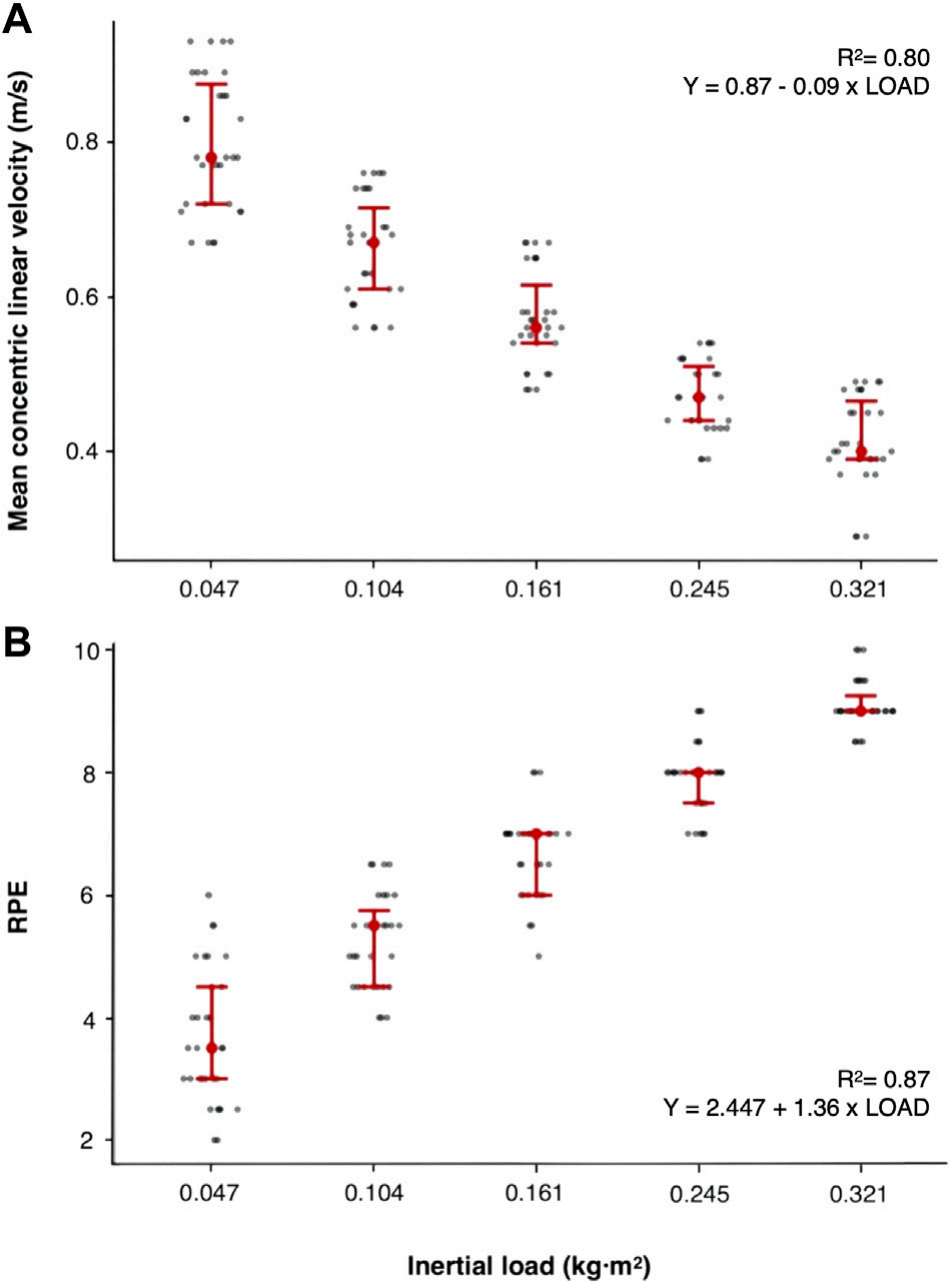
**(A)** Flywheel Load-Velocity profile, and **(B)** Flywheel Load-RPE profile during the flywheel squat exercise.

Participants attended to our laboratory in six occasions. All participants completed all the protocols, including four familiarisation sessions, and two testing sessions. The first four sessions were used as familiarisation sessions (Sabido et al., 2017). Familiarisation sessions, separated by 48 h, were performed to enlighten participants with the study procedures (i.e., flywheel operation and technique required and to familiarize them with the 5 loads used and RPE scale) and with the bilateral squat exercise proper technique and set-up in the flywheel device used (EPTE Inertial Concept, L’Alcudia, Spain) (Figure 1). Familiarization session 1 was used solely to educate participants on the correct exercise technique on the flywheel device employed (i.e., perform a maximal concentric contraction throughout the range of motion [ROM], to gently resist at the beginning of the eccentric phase, and then apply a maximum and brief eccentric contraction in the last third of the ROM). Familiarization sessions 2, 3 and 4 consisted of 6 sets of 6 repetitions with three different loads. After familiarisation, participants completed 2 testing sessions 1 week apart of the flywheel squat exercise, as detailed in the next section. The flywheel device was equipped with 6 combinable inertial wheels: 2 × 0.0095 kg m^2^, 2 × 0.0472 kg m^2^, 2 × 0.151 kg m^2^ and an integrated linear encoder (IMS measure system, L’Alcudia, Spain). The outcome measure was MCLV of each flywheel squat exercise set and RPE after each set. These measures were taken twice in each exercise mode (during experimental session 1 and 1 week after during experimental session 2) to check their reliability.

#### 2.2.1 Flywheel LV_profile_ test

During experimental sessions 1 and 2 an inertial progressive load test was performed to determine the flywheel LV_profile_ and its reliability. Each participant performed 5 series of 6 repetitions of the flywheel squat exercise with different inertial loads (0.047, 0.104, 0.161, 0.245, 0.321 kg m^2^) selected in a counterbalanced and randomized order with a 5-min rest period between sets to avoid the load effect on neuromuscular fatigue (Sabido et al., 2017). Session 2 was used to check the reliability of the data collected during session 1. In each set, the first repetition was performed to initiate the movement and to print speed into the flywheel system and it was not considered for further analysis. During the next five repetitions, participants were requested to apply maximal effort (i.e., maximum possible concentric velocity) throughout the concentric muscular contraction (ranged from 90°-knee flexion to near full extension, 0º-knee flexion). At the end of this concentric action, the flywheel strap wound back due to inertial forces, which initiated the reversed eccentric action. Participants were instructed to resist gently at the beginning of the eccentric phase to apply a brief and concentrated eccentric contraction at the end of the ROM (at about 90º-knee flexion). To ensure that participants employed the same squat depth at each repetition, an adjustable tripod with a telemetric photocell (Microgate, Bolzano, Italy) was placed at the side of the flywheel. The telemetry photocell emitted a sound when the knees reached the individual set height (Maroto-Izquierdo et al., 2020). MCLV of each set, estimated from the mean concentric velocity of the five maximum repetitions performed in each set, was collected for further analysis by the integrated linear encoder (2,000 Hz sampling rate, IMS measuring system, Spain). A 5-min rest period was provided between sets. A warm-up of 5-min cycling, 25 repetitions of skipping, 25 repetitions of butt kicks, and one set of the free-weight back squat at ∼8 RM load preceded the test.

#### 2.2.2 RPE assessment

In addition, during the 5-min rest period RPE was collected. 30 s after the end of each exercise set, participants were asked to rate their perceived exertion on a 0–10 RPE scale previously valid for resistance training (Lagally and Robertson, 2006) but not specific for flywheel exercise, where 10 was maximal perceived effort. The question they were asked was always the same: “How intense was the set?”. Participants were asked to mark their perceived exertion on an unnumbered visual analog scale. All participants were previously familiarized with the 0–10 RPE scale and they had previous experience on subjective effort reports.

### 2.3 Statistical analysis

All variables were expressed as a mean and standard deviation and were analyzed using a statistical package (R version 4.0.3, The R Foundation for Statistical Computing). Reliability between variables across experimental sessions was assessed by the intraclass correlation coefficient (ICC). Where values below 0.5 indicate poor reliability, between 0.5 and 0.75 moderate reliability, between 0.75 and 0.9 good reliability, and any ICC value above 0.9 indicates excellent reliability (Koo and Li, 2016). ICC values are expressed as the average value of the ICC of all loads. Pearson correlation coefficient was used to show the correlation between variables. Where values below 0.1 indicate negligible correlation, between 0.1 and 0.39 weak correlation, between 0.4 and 0.69 moderate correlation, between 0.7 and 0.89 strong correlation, and any R value above 0.9 indicates very strong correlation (Schober et al., 2018). Linear regressions were established in order to determinate the predictive equations considering load like independent variable, while MCLV and RPE were dependent variables; and secondly, considering MCLV as independent variable and RPE as a dependent variable. Significant differences were established at *p* < 0.05.

## 3 Results

In all cases, the assumptions of independence, homoscedasticity, normality and linearity were met. No significant differences (*p* > 0.05) were observed between the MCLV of the five maximum repetitions performed for each load. In addition, the average MCLV of the five repetitions performed with each load showed no significant differences between participants (*p* > 0.05). Descriptive data of MCLV and RPE for each load and testing day are shown in Table 1. The ICC between sessions showed values above 0.9 in all the variables studied (ICC = 0.94 in MCLV, and ICC = 0.97 in RPE).

**TABLE 1:** Mean ± SD of MCLV and RPE for each load and testing day.

	0.047 kg m^2^	0.104 kg m^2^	0.161 kg m^2^	0.245 kg m^2^	0.321 kg m^2^
Testing day 1
MCLV (m/s)	0.79 ± 0.10	0.66 ± 0.08	0.56 ± 0.07	0.47 ± 0.05	0.41 ± 0.06
RPE	3.66 ± 1.11	5.24 ± 0.77	6.66 ± 0.71	7.90 ± 0.57	9.11 ± 0.40
Testing day 2
MCLV (m/s)	0.80 ± 0.09	0.66 ± 0.07	0.57 ± 0.06	0.47 ± 0.05	0.41 ± 0.06
RPE	3.74 ± 1.04	5.26 ± 0.66	6.73 ± 0.70	7.92 ± 0.05	9.10 ± 0.40

Note: Values are means ± SD.

Abbreviations: MCLV, mean concentric linear velocity; RPE, Rate of Perceived Effort (between 0 and 10 points).

Significant correlations (*p* < 0.01) were found between load and MCLV, and load and RPE (Figures 1A,B, respectively). Indeed, the linear regression performed in order to predict the average MCLV as a function of the load used, LV_profile_, provided a significant (*p* < 0.01) model with *R* ^2^ = 0.80 (SE: 0.013) and an equation *y* = 0.87–0.09 × LOAD (Figure 1A). Similarly, a significant (*p* < 0.01) correlation model was observed when RPE (*R* ^2^ = 0.87; SE: 0.135; y = 2.447 + 1.36 x LOAD) was correlated with load (Figure 1B).

**FIGURE 2:**
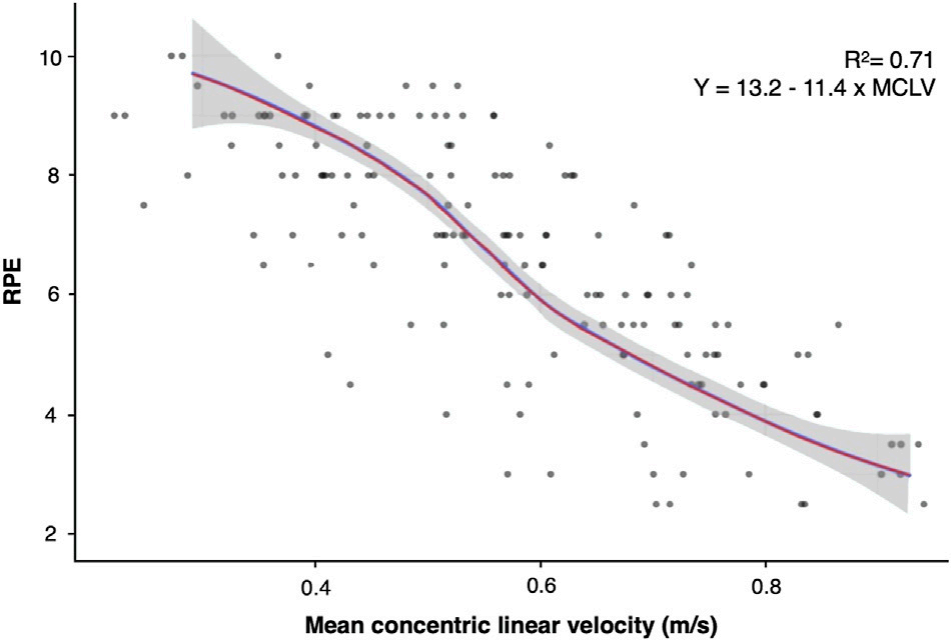
Regression plots between velocity and RPE.

Additionally, a Pearson’s correlation coefficient analysis was performed to determine the relationship between MCLV and RPE. A significant correlation (*p* < 0.01) was found between MCLV and RPE (Figure 2). Indeed, the linear regression performed to predict RPE as a function of the MCLV provided a significant (*p* < 0.01) model with *R* ^2^ = 0.71 (SE: 0.358) and an equation y = 13.2–11.4 x MCLV for RPE (Figure 2).

## 4 Discussion

The present study examined the relationship between inertial load and MCLV in the flywheel squat exercise. Additionally, this study aimed to correlate MCLV and RPE responses collected after preforming the flywheel squat exercise with different loads in physically active men. MCLV in the flywheel squat exercise has demonstrated to decrease while inertial load increases showing a significant load-velocity correlation (Figure 1) and significant model coefficients for each load (Table 1). These results, together with the high reliability observed between experimental session 1 and 2 have demonstrated that the control of MCLV during flywheel training can be proposed as a valid method to quantify load and to individualize the prescription of flywheel training. In addition, RPE responses have demonstrated significant correlations with load and velocity (Figures 1B, 2). Therefore, from the present results of this study, the flywheel LV_profile_ is an effective and reliable approach to categorize efforts during flywheel exercise and to assess training-induced effects. However, in the absence of a device that allows the measurement of MCLV, the control of the individual’s RPE can be a valid and reliable approach to quantify flywheel training.

In accordance with the force–velocity relationship of *in vivo* skeletal muscle studies (Hill, 1938), it has been shown that peak concentric velocity decreases while intensity increases during traditional weight training (Weakley et al., 2020). Similarly, McErlain and Beato (McErlain-Naylor and Beato, 2020) have observed that concentric and eccentric angular velocity during flywheel squats also decreased while inertial load increased. Concluding that low inertias may be well suited to stimulating a training-induced rightward shift of the force–velocity curve, whereas higher inertias may be better suited to stimulating an upward shift (McErlain-Naylor and Beato, 2020). Therefore, as Carroll et al. (Carroll et al., 2019) proposed, mean concentric velocity could be used as an avenue of intensity prescription for practitioners (Carroll et al., 2019; McErlain-Naylor and Beato, 2020; Spudić et al., 2020; Worcester et al., 2020). Spudić and others (Spudić et al., 2020) have recently demonstrated the reliability of the LV_profile_ outcome measures using the flywheel squat exercise with inertial loads ranged from 0.025 to 0.25 kg m^2^ (showing a significant correlation between force and mean velocity; *R* ^2^ = 0.96), which are in line with our results. Thus, the LV_profile_ has been lately recommended a valid and feasible way to prescribe flywheel exercise intensity and to assess training-induced effects (Maroto-Izquierdo et al., 2021). However, in daily practice, the most common parameters used to monitor flywheel exercise intensity are the moment of inertia (i.e., inertia load–the number and combination of wheels) and the power outputs (i.e., concentric and eccentric) (Maroto-Izquierdo et al., 2021). Even though monitoring of concentric and eccentric velocity have been suggested to effectively prescribe exercise intensity and should be preferred to power (Carroll et al., 2019; McErlain-Naylor and Beato, 2020; Beato et al., 2021; Maroto-Izquierdo et al., 2021), their accurate monitoring is quite complicated and it requires the use of advanced technologies (e.g., 3D motion capture or integrate linear encoders) that are not commonly available to practitioners.

This is the first study that analyzed the LV_profile_ during flywheel exercise using an integrated linear encoder to measure MCLV. In addition, kinematic variables such as MCLV might be crucial to determine differences between both concentric and eccentric contractions and also to identify the real eccentric-overload achieved and the mechanical tension experienced between different load conditions (Carroll et al., 2019; Núñez et al., 2020). Impulse (N·s^−1^) seems to be the key variable to control all these variables during flywheel training. Indeed, it has been previously reported that the eccentric-overload achieved during the flywheel squat was primarily a product of impulse characteristics (Carroll et al., 2019). Furthermore, we must be aware that the angular velocity/impulse measured at the level of the axis of rotation by rotatory encoders is not accurate enough (Weakley et al., 2019). Its lack of precision induces an asynchrony between what happens at the level of the axis of rotation and what happens at the level of the participant (Sabido et al., 2020b; Núñez et al., 2020). This is due to the difficulty in differentiating between concentric and eccentric actions since the inertia wheel only changes the direction of rotation between repetitions (Weakley et al., 2019; Bollinger et al., 2020). And also, as preliminary research has shown, rotary encoders function as a low-pass filter (i.e., smoothing the oscillations of high-frequency velocity actions) (Yan and Zhang, 2017), which is even accentuated with higher inertial loads, smoothing the instantaneous velocity record (Rojas-Delgado et al., 2019). Hence, implementing load quantification with the measurement of the linear velocity enables us to control the MCLV not only during the concentric phase but also during the eccentric one, providing more reliable and accurate data. Therefore, its implementation should be considered in flywheel training (Carroll et al., 2019). Given the inherent reliance of flywheel training on concentric output, prescribing intensities based on inertial load alone could become problematic in a training program that requires the prescription of specific training loads and monitoring. To solve this problem, this study has demonstrated the reliability and validity of the measurement of concentric linear velocity during the flywheel squat exercise in participants previously familiarized with this technology (inter-session reliability was 0.91 for MCLV). These results are in line with previous studies in which the reliability of concentric velocity at different intensities during flywheel exercise was studied (Spudić et al., 2020). Therefore, the results of this study propose the use of MCLV as a reliable intensity marker and a valid tool for exercise intensity prescription during flywheel training.

In addition, this study compared MCLV and inertial loads with the RPE responses in physically active men reporting that significant linear relationships between these parameters exist. When RPE was correlated with inertial loads, a strong (*R* ^2^ = 0.87) linear regression was found, i.e., equation: y = 2.447 + 1.36 × LOAD (Figure 2). The strength of this relationship is important for users because it shows that practitioners could use RPE to quantify the effort of their athletes (internal load) instead of using exclusively the moment of inertia used (external load) during a flywheel exercise. Moreover, this study reported a linear regression between RPE and MCLV, which was significant (*p* < 0.01) and strong *R* ^2^ = 0.71 (equation y = 13.2–11.4 × MCLV). This information is also very important, since velocity-based training is a very popular method in strength and conditioning, and it has been recently introduced into flywheel training as well (McErlain-Naylor and Beato, 2020; Maroto-Izquierdo et al., 2021). However, practitioners can struggle to monitor linear velocity in an applied setting (because of the necessity of using cameras or linear encoders) since rotatory encoders that are generally incorporated in flywheel devices are not suitable for this aim (Muñoz-López et al., 2022). Therefore, the use of RPE during daily monitoring could be a valid alternative to monitor flywheel exercise concentric velocity. The use of RPE for training load monitoring is also supported by its high reliability such as ICC = 0.93 (testing day 1 vs. testing 2), which highlights the capacity of familiarized users to report consistent perceived scores in different training sessions–for such a reason, RPE could be used to individualize the prescription of flywheel training (Beato et al., 2021). Although what just said about the use of this internal load parameter, RPE cannot replace completely the direct evaluation of MCLV using cameras or linear encoders as well as the knowledge of inertial loads and, therefore, it should be generally used as an additional parameter for training load monitoring (McErlain-Naylor and Beato, 2020; Maroto-Izquierdo et al., 2021).

### 4.1 Limitations and future perspectives

This study is not without limitations, firstly, a sample of sports science postgraduate male students was enrolled, therefore future studies should evaluate whether the results found in this study can be applied to other sport population such as professional athletes. Moreover, this study did not include female participants, therefore future research is needed to verify what was reported in this study such as the regressions models before that they are used with different populations. Secondly, inasmuch as there is not a validated RPE scale for flywheel exercise, the 0–10 RPE scale previously validated for weight training was used in this study. Therefore, future studies are warren to validate a flywheel exercise RPE scale. Finally, this study has verified the validity and reliability of the use of MCLV to calculate the LV_profile_ as well as to assess participants’ effort during the flywheel squat exercise, but no information is currently available on the eccentric velocity. Since eccentric velocity is a key aspect of flywheel training, future research is needed to verify if its monitoring is suitable in sport.

## 5 Conclusion

The findings of this study indicates that MCLV values decrease while inertial load increases during the flywheel squat exercise, showing high reliability and a significant load-velocity correlation. Collectively, these findings suggest that MCLV may be proposed as a valid method to quantify load and to individualize the prescription of flywheel training, which also could be used to assess training-induced effects. In addition, the strong correlation between RPE load and RPE and MCLV suggests the control of the individual’s RPE as a valid and reliable avenue to quantify flywheel training.

## Data Availability

The raw data supporting the conclusions of this article will be made available by the authors, without undue reservation.
